# Diverse evolutionary trajectories of *Klebsiella pneumoniae* carbapenemase: unraveling the impact of amino acid substitutions on β-lactam susceptibility and the role of avibactam in driving resistance

**DOI:** 10.1128/msystems.00184-25

**Published:** 2025-03-11

**Authors:** Jie Wei, Jinyu Huang, Chunhong Zou, Shimei Shen, Barry N. Kreiswirth, Ailong Huang, Shifeng Huang, Liang Chen, Deqiang Wang, Siqiang Niu

**Affiliations:** 1 Department of Laboratory Medicine, The First Affiliated Hospital of Chongqing Medical University, Yuzhong, Chongqing, China; 2 The Key Laboratory of Molecular Biology of Infectious Diseases designated by the Chinese Ministry of Education Chongqing Medical University, Yuzhong, Chongqing, China; 3 Department of Clinical Laboratory, Zhuhai People’s Hospital (Zhuhai Clinical Medical College of Jinan University), Zhuhai, China; 4 College of Laboratory Medicine, Chongqing Medical University, Yuzhong, Chongqing, China; 5 Department of Clinical Laboratory, University-Town Hospital of Chongqing Medical University 568864 , Chongqing, China; 6 Center for Discovery and Innovation Hackensack Meridian Health, Nutley, New Jersey, USA; 7 Department of Pharmacy Practice, School of Pharmacy and Pharmaceutical Sciences University at Buffalo, Buffalo, New York, USA; 8 Western (Chongqing) Institut for Digital-Intelligent Medicine, Chongqing National Biomedicine Industry Park, Chongqing, China; University of California San Diego, La Jolla, California, USA

**Keywords:** KPC (*Klebsiella pneumoniae *carbapenemase), phylogenetic reconstruction, ceftazidime-avibactam, evolution, resistance

## Abstract

*Klebsiella pneumoniae* carbapenemases (KPCs) have evolved into over 245 distinct variants, with over one-third of variants exhibiting reduced susceptibility to ceftazidime-avibactam, while the underlying selection mechanisms remain elusive. To better elucidate these resistant phenotypes, we cloned 33 clinically described KPC variants (from KPC-2 to KPC-36) and 8 artificially created variants into a common plasmid vector and assessed their impact on β-lactam susceptibility. Strains expressing KPC-14, KPC-28, and KPC-31 exhibited increased resistance to ceftazidime and ceftazidime-avibactam but decreased resistance to carbapenems. We further studied the catalytic mechanism of β-lactam hydrolysis by KPC-4, KPC-14, KPC-15, KPC-16, KPC-21, KPC-25, KPC-28, KPC-31, and the ancestral KPC-2 and KPC-3 enzymes. Antimicrobial susceptibility test, enzyme kinetics, and molecular modeling revealed diverse selective pressures, including but not limited to aztreonam and ceftriaxone, driving KPC evolution, with ceftazidime playing a central role. Substitutions within the KPC hydrolytic active sites notably reduced the inhibitory effect of avibactam on KPC, demonstrated by isothermal titration calorimetry analysis, resulting in enhanced hydrolysis of ceftazidime by enzyme kinetics. This highlights that avibactam may serve as an additional driving force in KPC evolution.

**IMPORTANCE:**

The rapid evolution of KPC carbapenemases, including resistance to ceftazidime-avibactam, threatens the effectiveness of last-resort antibiotics against *Klebsiella pneumoniae* infections, necessitating understanding of of the underlying selection pressures. This study investigates the evolutionary mechanisms driving KPC diversification and resistance to ceftazidime-avibactam, providing crucial information for developing effective strategies to combat carbapenem-resistant *Klebsiella pneumoniae* (CRKP) infections and preserve antibiotic efficacy.

## INTRODUCTION

*Klebsiella pneumoniae* carbapenemases (KPCs), encoded by the *bla*_KPC_ gene and belonging to class A β-lactamases, stand out as the most common carbapenemases globally, causing carbapenem-resistant *Enterobacterales* (1). Remarkably, numerous novel KPC variants conferring ceftazidime-avibactam (CAZ-AVI) resistance have emerged in the last 5 years, very likely due to clinical introduction of CAZ-AVI, representing 30%–40% of 245 distinct KPC variants cumulatively documented in the Beta-Lactamase Database—Structure and Function website (http://www.bldb.eu/Enzymes.php). KPCs, through their serine active site (S70), hydrolyze nitrocefin, cephalothin, and cephaloridine efficiently; carbapenems and cefotaxime moderately; and cefoxitin and CAZ poorly (2–5). KPC-33 (6) and KPC-31 (7), single amino acid variants (D179Y) of KPC-2 and KPC-3, respectively, confer CAZ-AVI resistance while simultaneously reducing carbapenem resistance and are frequently reported clinically. Variants such as KPC-94 (LN169-170H), KPC-95 (D179Y/A172T) (8), and KPC-41 (269–270 PNK insertions) (9) exhibit high CAZ-AVI minimum inhibitory concentrations (MICs) (16–128 mg/L).

Despite these findings, the underlying selection mechanisms responsible for the emergence of CAZ-AVI resistance remain unclear. Given the potential for novel KPC variants to compromise the efficacy of new β-lactamase inhibitors (BLIs) in safeguarding β-lactam antibiotics, our investigation about the systematic evolution of KPC variants sought to emphasize the key factors driving KPC diversification and selection, as well as to underscore the complex interplay between antibiotic exposure and the emergence of resistance. We reconstructed *bla*_KPC_ gene variants, assessed their impact on susceptibility to β-lactam antibiotics in isogenic strains carrying different KPC variants using the standard broth microdilution method, and compared the enzyme kinetic parameters of key KPC variants to gain mechanistic insights. Subsequently, we employed molecular docking modeling and isothermal titration calorimetry to examine the impact of mutant KPC amino acid residues on the interaction between KPC enzymes and AVI. This comprehensive study unveiled the primary drivers behind the evolution of KPC variants.

## RESULTS

### Phylogenetic analysis of KPC variants

**Fig 1:**
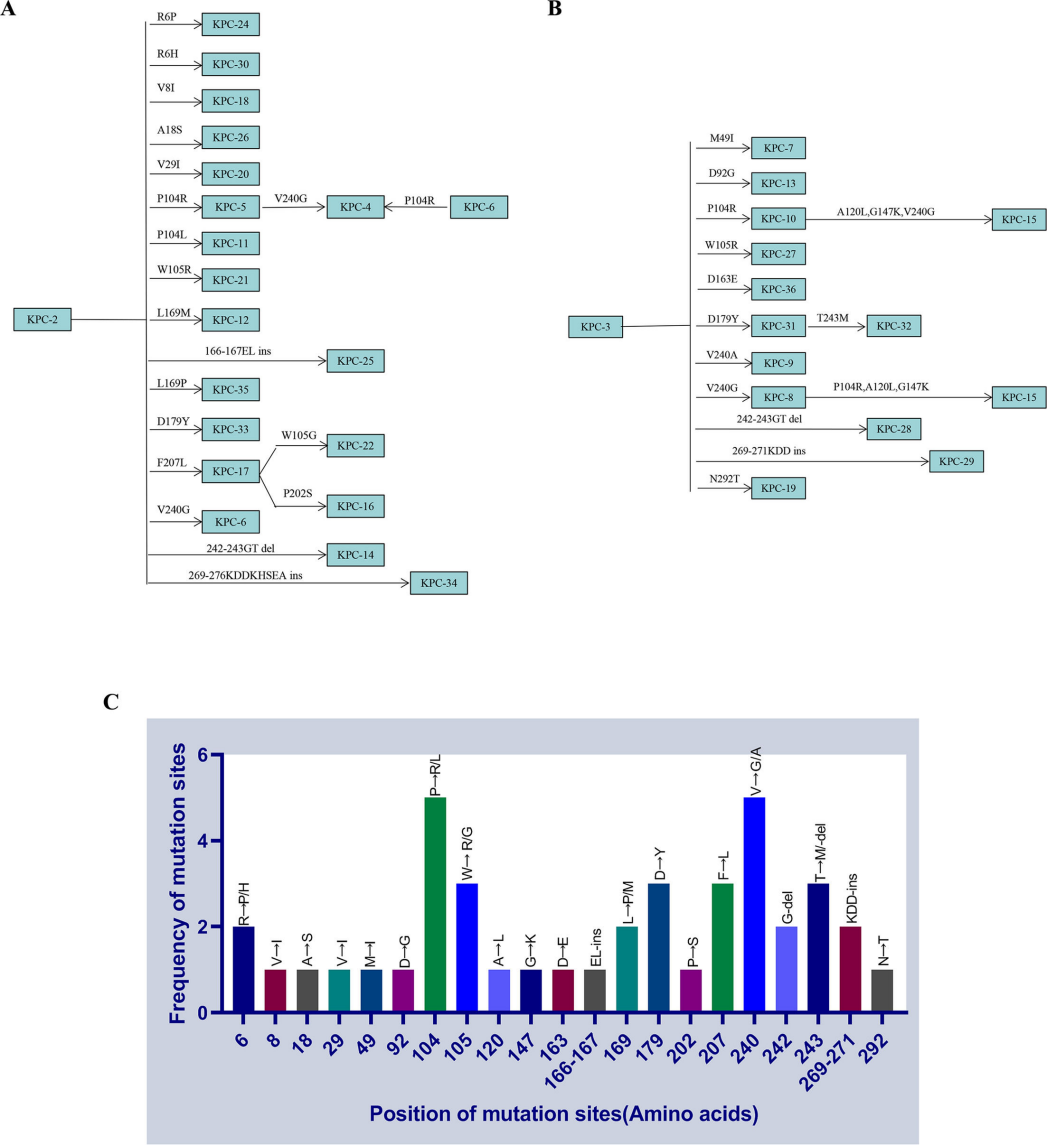
Phylogenetic ancestral reconstruction and statistical frequency of mutation sites of KPC variants. (A) KPC-2 cluster. (B) KPC-3 cluster. The ancestral states were computed using the MEGA-X program, employing parsimony methods. The branches represent the amino acid changes acquired in comparison to the ancestor’s sequence. (C) Statistical frequency of mutation sites in natural KPC mutants (KPC-2 to KPC-36). "R→P/H" indicates that the amino acid “R” was replaced by either the amino acid “P” or “H.” del, deletion; ins, insertion.

At the inception of this project in August 2018, KPC variants 2–36 had been documented, and a maximum-likelihood (ML) phylogenetic tree of *bla*_KPC-2_ to *bla*_KPC-36_ was constructed (Fig. S1). The reconstruction of phylogenetic ancestors revealed that the diversification of *bla*_KPC-2_ and *bla*_KPC-3_ nodes primarily involved single nonsynonymous mutations, accounting for 22 non-synonymous changes out of a total of 32 alterations (Fig. 1). A noteworthy distinguishing characteristic between *bla*_KPC-3_ and *bla*_KPC-2_ is a non-synonymous C814T nucleotide substitution, resulting in the H274Y mutation. It is worth noting that 13 out of 19 new variants derived directly from *bla*_KPC-2_, each carrying a single non-synonymous mutation (Fig. 1A). Similarly, 9 of 13 new variants from the *bla*_KPC-3_ node exhibited the same pattern (Fig. 1B). Positions P104, W105, D179, F207, V240, G242, and T243 demonstrated mutation frequencies of at least three variants, mainly located in the Ω-loop (R164–D179) and loops 237–243 (Fig. 1C). Despite the ancestral *bla*_KPC_ genes having emerged two decades ago, over 91% (225 of 245) of the *bla*_KPC_ variants were discovered from 2015, the year CAZ-AVI was marketed, suggesting a recent and accelerated diversification (10).

### Site-directed mutagenesis and resistance profiling of KPC variants in isogenic strains

To investigate the effects of amino acid substitutions on antibiotic susceptibility, isogenic *Escherichia coli* DH10B strains expressing *bla*_KPC_ variants 2–36 and eight additional non-natural mutations were constructed. This approach allowed correlation of resistance changes with specific mutations. Most KPC-2 cluster variants showed lower MICs for piperacillin, piperacillin-tazobactam, cefazolin, cefuroxime, imipenem, and meropenem (MEM) compared to the KPC-2 reference strain (Table 1). Only a few variants, such as KPC-6 and KPC-30, exhibited increased MICs for piperacillin-tazobactam, imipenem, and meropenem. However, the KPC-3 cluster did not follow a similar pattern; only a few variants, such as KPC-27, KPC-28, KPC-29, and KPC-31 exhibited decreased MICs of piperacillin, cefazolin, cefuroxime, ceftriaxone, cefepime, aztreonam, and the carbapenems (Table 2). CAZ resistance profiles differed significantly. Six variants within the KPC-2 cluster (namely, KPC-4, KPC-5, KPC-6, KPC-14, KPC-33, and KPC-35) but 16 variants within the KPC-3 cluster (excluding KPC-13, KPC-19, KPC-27, KPC-29, and KPC-36) exhibited at least fourfold higher CAZ MICs than the KPC-2 reference strain (Table 3).

**TABLE 1:** MICs of different β-lactam antibiotics in the context of bacteria expressing various KPC-2 cluster variants

Change introduced	Variant	MIC (mg/L)^*a*^											
		PIP	TZP	CZO	CXM	CAZ	CAZ-AVI	CRO	FEP	ATM	ATM-AVI	IPM	MEM
–	KPC-2	>256	256	256	>256	8	0.25	128	4	32	0.125	1	0.25
P104R, V240G	KPC-4	256	8	128	>256	256	1	>256	8	>256	0.25	1	0.25
P104R	KPC-5	>256	16	128	>256	64	0.5	>256	4	>256	0.125	1	0.25
V240G	KPC-6	>256	>256	256	>256	32	0.5	>256	16	>256	0.125	2	0.5
P104L	KPC-11	256	8	128	128	2	0.0625	4	1	8	<0.0156	0.5	0.25
L169M	KPC-12	8	2	32	64	1	0.0625	2	0.5	4	<0.0156	0.5	<0.0312
242–243 GT del	KPC-14	32	2	256	64	256	16	4	8	16	<0.0156	0.25	<0.0312
P202S, F207L	KPC-16	256	16	256	>256	2	<0.0156	128	4	16	<0.0156	1	0.25
F207L	KPC-17	256	32	256	>256	1	0.0625	32	0.5	8	<0.0156	1	0.125
V8I	KPC-18	>256	64	256	>256	4	0.0625	16	1	16	<0.0156	1	0.125
V29I	KPC-20	>256	256	>256	>256	4	0.0312	64	4	32	0.0312	1	0.5
W105R	KPC-21	8	1	16	32	1	0.0625	0.5	<0.125	32	<0.0156	0.25	<0.0312
W105G, F207L	KPC-22	128	16	64	64	0.25	<0.0156	1	0.25	4	0.0312	0.5	<0.0312
R6P	KPC-24	>256	256	256	>256	2	0.25	>256	4	32	0.0312	1	0.5
166–167EL ins	KPC-25	4	4	4	8	4	2	0.5	<0.125	0.5	0.0625	0.5	<0.0312
A18S	KPC-26	>256	64	256	256	2	0.0625	4	1	16	<0.0156	1	0.0625
R6H	KPC-30	>256	>256	256	>256	8	0.25	>256	4	32	0.0312	2	0.5
D179Y	KPC-33	4	1	2	8	32	0.125	2	0.5	0.25	<0.0156	0.125	<0.0312
L169P	KPC-35	4	2	8	32	32	0.0625	2	2	0.5	0.0312	0.125	<0.0312
	*E. coli* DH10B	2	2	2	4	0.25	<0.125	<0.125	<0.125	<0.125	0.0312	0.25	<0.0312
	*Kpn* with KPC-3	>256	256	>256	>256	>256	2	>256	>256	>256	0.125	16	>4

^*a*^ The MICs are the medians of three or more values obtained or an MIC value obtained more than once. ATM, aztreonam; ATM-AVI, aztreonam-avibactam; CAZ, ceftazidime; CAZ-AVI, ceftazidime-avibactam; CRO, ceftriaxone; CXM, cefuroxime; CZO, cefazolin; FEP, cefepime; IPM, imipenem; MEM, meropenem; PIP, piperacillin; TZP, piperacillin-tazobactam. *E. coli* DH10B cells containing the empty vector and *Klebsiella pneumoniae* producing KPC-3 were used as the negative control and positive control CRE strains, respectively.

**TABLE 2:** MICs of different β-lactam antibiotics in the context of bacteria expressing various KPC-3 cluster variants

Change introduced	Variant	MIC (mg/L)^*a*^											
		PIP	TZP	CZO	CXM	CAZ	CAZ-AVI	CRO	FEP	ATM	ATM-AVI	IPM	MEM
–	KPC-3	>256	>256	256	>256	64	0.5	128	8	64	0.0625	2	0.25
M49I	KPC-7	>256	16	256	>256	64	1	>256	2	>256	0.0625	1	>4
V240G	KPC-8	256	128	>256	>256	128	0.5	128	16	>256	<0.0156	1	0.25
V240A	KPC-9	>256	16	256	>256	32	0.0625	64	1	32	<0.0156	1	0.0625
P104R	KPC-10	128	4	128	256	64	0.125	4	2	128	<0.0156	1	0.25
D92G	KPC-13	256	16	128	>256	8	<0.0156	4	2	8	<0.0156	1	0.25
P104R, A120L, G147K, V240G	KPC-15	16	1	128	32	64	4	128	4	128	0.5	0.5	<0.0312
N292T	KPC-19	128	8	256	128	8	0.0625	8	1	16	<0.0156	1	0.125
W105R	KPC-27	16	1	32	64	4	0.0312	0.25	<0.125	64	0.0312	0.5	<0.0312
242–243 GT del	KPC-28	16	1	128	16	64	>16	1	2	8	<0.0156	0.125	<0.0312
269–271 KDD ins	KPC-29	16	2	64	64	8	0.125	1	2	4	<0.0156	1	<0.0312
D179Y	KPC-31	8	4	4	16	128	16	4	2	1	0.0625	0.5	<0.0312
D179Y, T243M	KPC-32	>256	64	256	>256	32	0.5	>256	8	64	0.0312	1	0.25
D163E	KPC-36	256	16	128	128	8	0.125	2	2	16	0.0312	1	0.125
	*E. coli* DH10B	2	2	2	4	0.25	<0.125	<0.125	<0.125	<0.125	0.0312	0.25	<0.0312
	Kpn with KPC-3	>256	256	>256	>256	>256	2	>256	>256	>256	0.125	16	>4

^*a*^ The MICs are the medians of three or more values obtained or an MIC value obtained more than once. ATM, aztreonam; ATM-AVI; aztreonam-avibactam; CAZ, ceftazidime; CAZ-AVI, ceftazidime-avibactam; CRO, ceftriaxone; CXM, cefuroxime; CZO, cefazolin; FEP, cefepime; IPM, imipenem; MEM, meropenem; PIP, piperacillin; TZP, piperacillin-tazobactam. *E. coli* DH10B cells containing the empty vector and *Klebsiella pneumoniae* producing KPC-3 were used as the negative control and positive control CRE strains, respectively.

**TABLE 3:** MICs of different β-lactam antibiotics in the context of bacteria expressing eight non-natural KPC variants

Change introduced	Variant	MIC (mg/L) ^*a*^											
		PIP	TZP	CZO	CXM	CAZ	CAZ-AVI	CRO	FEP	ATM	ATM-AVI	IPM	MEM
–	KPC-3	>256	>256	256	>256	64	0.5	128	8	64	0.0625	2	0.25
A125V	KPC-3^A125V^	256	4	128	>256	64	4	32	2	16	0.125	1	0.0625
166–167EL del	KPC-3^166-167EL del^ (KPC-66)	4	2	2	16	32	1	2	1	0.5	0.0625	0.5	<0.0312
166–167EL ins	KPC-3^166-167EL ins^ (KPC-53)	8	4	8	8	32	1	2	0.5	4	0.0312	0.25	<0.0312
A201V, V240G	KPC-8^A201V^	>256	16	256	>256	256	2	>256	32	>256	0.0625	1	0.25
P104R, A120L, G147K, A201V, V240G	KPC-15^A201V^	16	1	64	256	64	2	2	1	64	0.0312	0.25	<0.0312
P104R, A120L, G147K, L162M, V240G	KPC-15^L162M^	16	0.5	128	32	64	4	128	2	64	0.5	0.5	<0.0312
A177E, D179Y	KPC-31^A177E^	8	2	4	16	128	16	1	1	0.5	0.125	0.5	<0.0312
L10P, A177E, D179Y	KPC-31^L10P, A177E^	4	2	2	16	64	8	0.25	0.25	<0.125	0.125	0.25	<0.0312
	*E. coli* DH10B	2	2	2	4	0.25	<0.125	<0.125	<0.125	<0.125	0.0312	0.25	<0.0312
	Kpn with KPC-3	>256	256	>256	>256	>256	2	>256	>256	>256	0.125	16	>4

^*a*^ The MICs are the medians of three or more values obtained or an MIC value obtained more than once. ATM, aztreonam; ATM-AVI; aztreonam-avibactam; CAZ, ceftazidime; CAZ-AVI, ceftazidime-avibactam; CRO, ceftriaxone; CXM, cefuroxime; CZO, cefazolin; FEP, cefepime; IPM, imipenem; MEM, meropenem. *E. coli* DH10B cells containing the empty vector and *Klebsiella pneumoniae* producing KPC-3 were used as the negative control and positive control CRE strains, respectively.

Notably, the KPC-3 cluster displayed substantially higher CAZ MIC_50_ and MIC_90_ values (32- and 16-fold, respectively) compared to the KPC-2 cluster, attributable to the single H274Y substitution (Table S3). Furthermore, increased CAZ resistance was linked to mutations P104R, L169P, D179Y, V240G, and a 242–243 GT deletions, especially in KPC-2 cluster variants, many of which are high-frequency mutations (Fig. 1C). While these mutations in KPC-3 did not increase the CAZ MIC, mutations at other locations significantly reduced the CAZ MIC. Notably, different mutations at the same site (P104L, L169M, and V240A) also resulted in reduced CAZ MICs. This suggests that these sites (P104R, L169P, D179Y, V240G, and a 242–243 GT deletions) are crucial for maintaining CAZ hydrolysis. Interestingly, the co-existence of two high-frequency substitutions, as demonstrated by instances such as KPC-4 with P104R and V240G, KPC-8 with V240G and H274Y, and KPC-31 with D179Y and H274Y, resulted in additive increases in CAZ resistance. Bacterial strains producing KPC-14, KPC-28, KPC-31, and KPC-3^A177E, D179Y^ exhibited a substantial 64-fold increase in resistance to CAZ-AVI.

A heatmap visualizing MIC data (Fig. S2) showed that KPC-4, KPC-5, KPC-6, and KPC-14 exhibited 2–5 log_2_-fold increases in CAZ resistance compared to KPC-2. This trend was similarly reflected in resistance to CAZ-AVI. Conversely, among KPC-3 variants, KPC-7, KPC-15, KPC-28, and KPC-31, and eight non-natural variants displayed 1–5 log_2_-fold increases in CAZ-AVI resistance, with slightly increased or even decreased CAZ resistance. Compared to the recipient strain carrying KPC-3, strains carrying KPC-3^A125V^, KPC-3^166-167EL del^, KPC-3^166-167EL ins^, KPC-3^A201V, V240G^, KPC-3^P104R, A120L, G147K, A201V, V240G^, KPC-3^P104R, A120L, G147K, L162M, V240G^, KPC-3^A177E, D179Y^, and KPC-3^L10P, A177E, D179Y^ mediated 8-, 2-fold, 2-, 4-, 4-, 8-, 32-, and 16-fold increases in MICs for CAZ-AVI, respectively (Table 3, Fig. S2). Unsurprisingly, KPC-3^166-167EL ins^ and KPC-3^166-167EL del^ were successively reported as KPC-53 and KPC-66 natural variants, and the results showed decreased CAZ resistance and even carbapenems but increased CAZ-AVI resistance (11, 12), which was consistent with our results. Collectively, these findings suggest that KPC-2 cluster variants enhance CAZ-AVI resistance by speeding up CAZ hydrolysis, while KPC-3 cluster variants do so by reducing AVI inhibition.

Subsequently, to determine if MIC differences correlated with KPC expression levels, immunoblot analysis was performed on KPC-2, KPC-3, KPC-4, KPC-14, KPC-15, KPC-16, KPC-21, KPC-25, KPC-28, and KPC-31 (Fig. S3A). Compared to KPC-2, the protein expression levels of KPC-4, KPC-14, KPC-16, KPC-21, and KPC-31 exhibited minor fluctuations within a range of 20%. Conversely, KPC-3 expression significantly increased over 50%, while KPC-25, KPC-15, and KPC-28 showed substantial decreases (−56.76%, −22.14%, and −11.06%, respectively). KPC-3 exhibited greater stability than other variants, while KPC-28 showed lower stability. Intriguingly, despite lower expression levels, KPC-15, KPC-25, and KPC-28 did not exhibit the lowest CAZ-AVI MICs. This suggests that CAZ-AVI resistance is mediated by factors beyond KPC expression levels, such as changes in protein conformation and stability resulting from mutations.

### Expression, purification, and steady-state kinetic characterization of KPC variants

Ten KPC proteins, excluding signal peptides (Fig. S3B), were expressed and purified. In a comparison of enzymatic activities between KPC-2 and KPC-3, the H274Y substitution more than halved the *K*_*m*_ values for ceftazidime (590.72 µM vs 233.85 µM) and aztreonam (2,398.45 µM vs 221.15 µM) while increasing the *K*_*m*_ for cefazolin (110.75 µM vs 368.15 µM). The *k*_cat_/*K*_*m*_ ratios for CAZ (0.0055 ± 0.0004 μM^−1^·s^−1^ vs 0.011 ± 0.0012 µM^−1^·s^−1^), aztreonam (0.005 ± 0.0003 µM^−1^·s^−1^ vs 0.036 ± 0.003 µM^−1^·s^−1^), and imipenem (0.29 ± 0.01 µM^−1^·s^−1^ vs 0.52 ± 0.034 µM^−1^·s^−1^) increased, aligning with the observed MIC values for these three drugs. In contrast, *k*_cat_/*K*_*m*_ ratios for piperacillin (0.0097 ± 0.0009 µM^−1^·s^−1^ vs 0.0048 ± 0.0004 µM^−1^·s^−1^), cefazolin (0.59 ± 0.041 µM^−1^·s^−1^ vs 0.17 ± 0.013 µM^−1^·s^−1^), cefepime (0.015 ± 0.0012 µM^−1^·s^−1^ vs 0.0089 ± 0.0004 µM^−1^·s^−1^), and meropenem (0.50 ± 0.05 µM^−1^·s^−1^ vs 0.23 ± 0.016 µM^−1^·s^−1^) decreased, while no significant changes were noted for four other antibiotics. Furthermore, the 242–243 GT deletions in KPC-14 significantly increased the *k*_cat_/*K*_m_ ratios for CAZ (0.33 ± 0.009 µM^−1^·s^−1^ vs 0.0055 ± 0.0004 µM^−1^·s^−1^) and cefepime (0.11 ± 0.004 µM^−1^·s^−1^ vs 0.015 ± 0.0012 µM^−1^·s^−1^) but decreased for carbapenems. Additionally, the 242–243 GT deletions also increased aztreonam (192.34 µM vs 2,398.45 µM) and cefepime (70.41 µM vs 310.48 µM) hydrolyses while diminishing carbapenem hydrolysis. KPC-28 (242–243 GT del/H274Y) showed a similar effect on CAZ (0.01 ± 0.0004 µM^−1^·s^−1^ vs 0.0055 ± 0.0004 µM^−1^·s^−1^) but not additively (Table 4). Intriguingly, the MIC value for CAZ was lower in KPC-28 compared to KPC-14, likely attributable to the inhibitory effect of KPC-28 expression (Fig. S3A). KPC-31 (D179Y) showed reduced CAZ hydrolysis (1,737.14 μM vs 233.85 µM) and *k*_cat_/*K*_*m*_ (0.0079 ± 0.00004 µM^−1^·s^−1^ vs 0.011 ± 0.0012 µM^−1^·s^−1^) compared to KPC-3, yet a higher CAZ-AVI MIC (0.5 mg/L vs 16 mg/L) (Table 2), suggesting reduced AVI inhibition of KPC-31 contributes to increased CAZ-AVI resistance.

**TABLE 4:** Kinetic parameters of antibiotic hydrolysis by KPC variants^*a*^

Parameter and antibiotic^*b*^	KPC-2	KPC-3	KPC-4	KPC-14	KPC-15	KPC-16	KPC-21	KPC-25	KPC-28	KPC-31
*K*_*m*_ (μM)										
MEM	16 ± 1	24 ± 1	16 ± 1	NH	168 ± 15	13 ± 0.6	48 ± 3	3,783 ± 353	NH	NH
IPM	98 ± 9	64 ± 3	96 ± 1	548 ± 46	104 ± 7	53 ± 4	19 ± 1	NH	563 ± 49.	3,508 ± 196
CAZ	590 ± 48	233 ± 19	213 ± 14	73 ± 1	251 ± 23	390 ± 28	NH	NH	280 ± 9	1,737 ± 135
ATM	2,398 ± 161	221 ± 10	763 ± 56	192 ± 16	336 ± 33	760 ± 48	283 ± 25	NH	NH	NH
FEP	310 ± 23	512 ± 48	277 ± 15	70 ± 5	371 ± 19	53 ± 3	277 ± 27	NH	115 ± 9	NH
PIP	793 ± 34	1,097 ± 84	231 ± 21	45 ± 0.5	86 ± 3	765 ± 68	NH	NH	61 ± 4	NH
CZO	110 ± 3	368 ± 16	113 ± 9	287 ± 15	645 ± 22	253 ± 12	437 ± 38	73 ± 5	680 ± 65	142 ± 7
*k*_cat_ (s^−1^)										
MEM	8 ± 0.5	5.7 ± 0.4	6.6 ± 0.4	NH	4.8 ± 0.01	1.9 ± 0.1	10 ± 1	4 ± 0.2	4.1 ± 0.3	1.1 ± 0.1
IPM	28 ± 2.	33 ± 0.7	22 ± 2	19 ± 1	7.5 ± 0.7	12 ± 1	1.8 ± 0.1	NH	3.8 ± 0.3	7.7 ± 0.4
CAZ	3.27 ± 0.27	2.7 ± 0.2	5.4 ± 0.04	24 ± 1	6.1 ± 0.5	0.04 ± 0.002	NH	NH	6.1 ± 0.4	4.7 ± 0.4
ATM	12.6 ± 0.6	7.9 ± 0.1	15 ± 0.8	2.8 ± 0.2	16 ± 1	7.7 ± 0.5	1.7 ± 0.1	NH	28 ± 2	9.1 ± 0.5
FEP	4.7 ± 0.1	4.5 ± 0.3	16 ± 0.3	7.6 ± 0.2	14 ± 0.7	2.3 ± 0.2	3 ± 0.1	NH	22 ± 1	3.2 ± 0.2
PIP	7.7 ± 0.5	5.2 ± 0.1	6.6 ± 0.4	1.08 ± 0.02	1.8 ± 0.2	4.1 ± 0.2	NH	NH	1.5 ± 0.1	1.02 ± 0.1
CZO	65 ± 5	61 ± 2	59 ± 4	27 ± 2	51 ± 5	72 ± 3	12 ± 0.9	7.3 ± 0.4	14 ± 1	1.30 ± 0.1
*k*_cat_/*K*_*m*_ (μM^−1^·s^−1^)										
MEM	0.50 ± 0.05	0.23 ± 0.016	0.41 ± 0.013	NH	0.30 ± 0.029	0.12 ± 0.005	0.61 ± 0.026	0.25 ± 0.017	0.26 ± 0.019	0.068 ± 0.003
IPM	0.29 ± 0.01	0.52 ± 0.034	0.23 ± 0.012	0.035 ± 0.002	0.076 ± 0.007	0.13 ± 0.010	0.018 ± 0.0016	NH	0.039 ± 0.003	0.078 ± 0.005
CAZ	0.0055 ± 0.0004	0.011 ± 0.0012	0.0091 ± 0.0003	0.33 ± 0.009	0.01 ± 0.0008	0.00007 ± 0.00001	NH	NH	0.01 ± 0.0004	0.0079 ± 0.00004
ATM	0.005 ± 0.0003	0.036 ± 0.003	0.0064 ± 0.0002	0.014 ± 0.0007	0.0067 ± 0.0003	0.0032 ± 0.00027	0.00071 ± 0.00002	NH	0.012 ± 0.0006	0.0038 ± 0.0003
FEP	0.015 ± 0.0012	0.0089 ± 0.0004	0.053 ± 0.003	0.11 ± 0.004	0.047 ± 0.002	0.016 ± 0.0003	0.0096 ± 0.0005	NH	0.07 ± 0.002	0.01 ± 0.0001
PIP	0.0097 ± 0.0009	0.0048 ± 0.0004	0.0084 ± 0.0002	0.023 ± 0.001	0.0022 ± 0.0001	0.0052 ± 0.0004	NH	NH	0.0019 ± 0.00015	0.0013 ± 0.00005
CZO	0.59 ± 0.041	0.17 ± 0.013	0.54 ± 0.036	0.094 ± 0.006	0.46 ± 0.01	0.66 ± 0.02	0.011 ± 0.01	0.065 ± 0.005	0.13 ± 0.005	0.011 ± 0.0004

^*a*^ ATM, aztreonam; CAZ, ceftazidime; CZO, cefazolin; FEP, cefepime; IPM, imipenem; MEM, meropenem; IPM, imipenem; NH, no detectable hydrolysis.

^*b*^ Data represent the means of three independent experiments.

### Molecular modeling of CAZ and AVI docking to KPC variants

**Fig 2:**
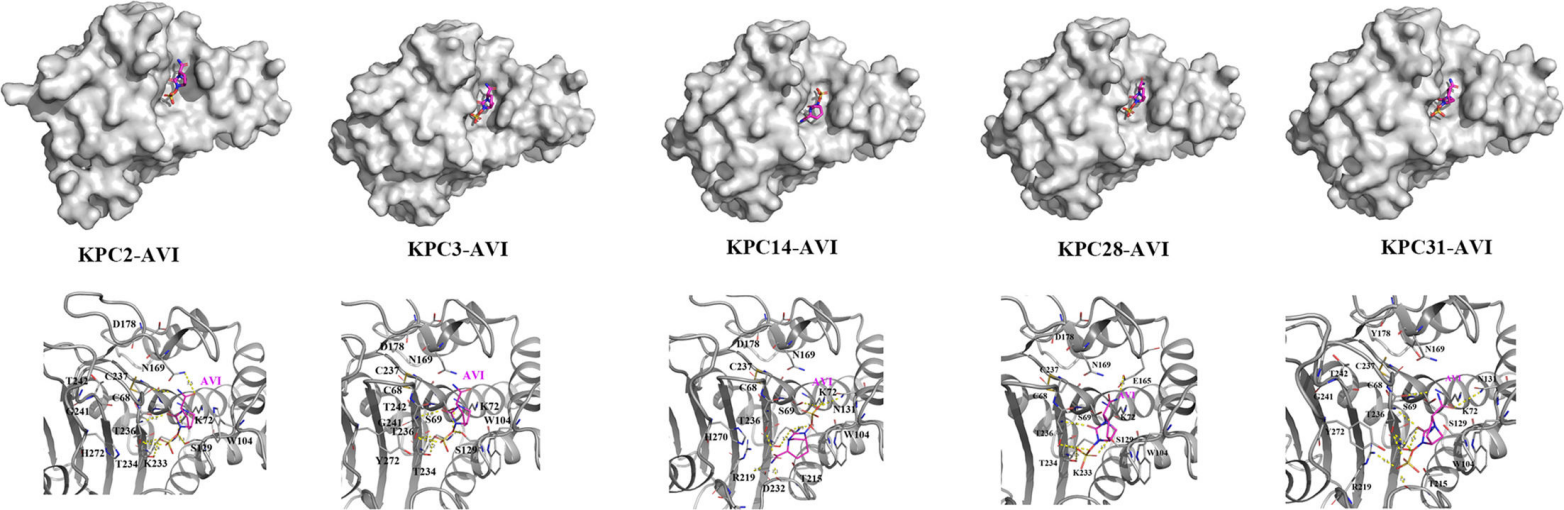
Models of interaction between KPC-2, KPC-3, KPC-14, KPC-28, and KPC-31 with AVI. Superimposing the crystal structure of KPC-2 in complex with AVI onto the homology models of KPC-3, KPC-14, KPC-28, and KPC-31.

**Fig 3:**
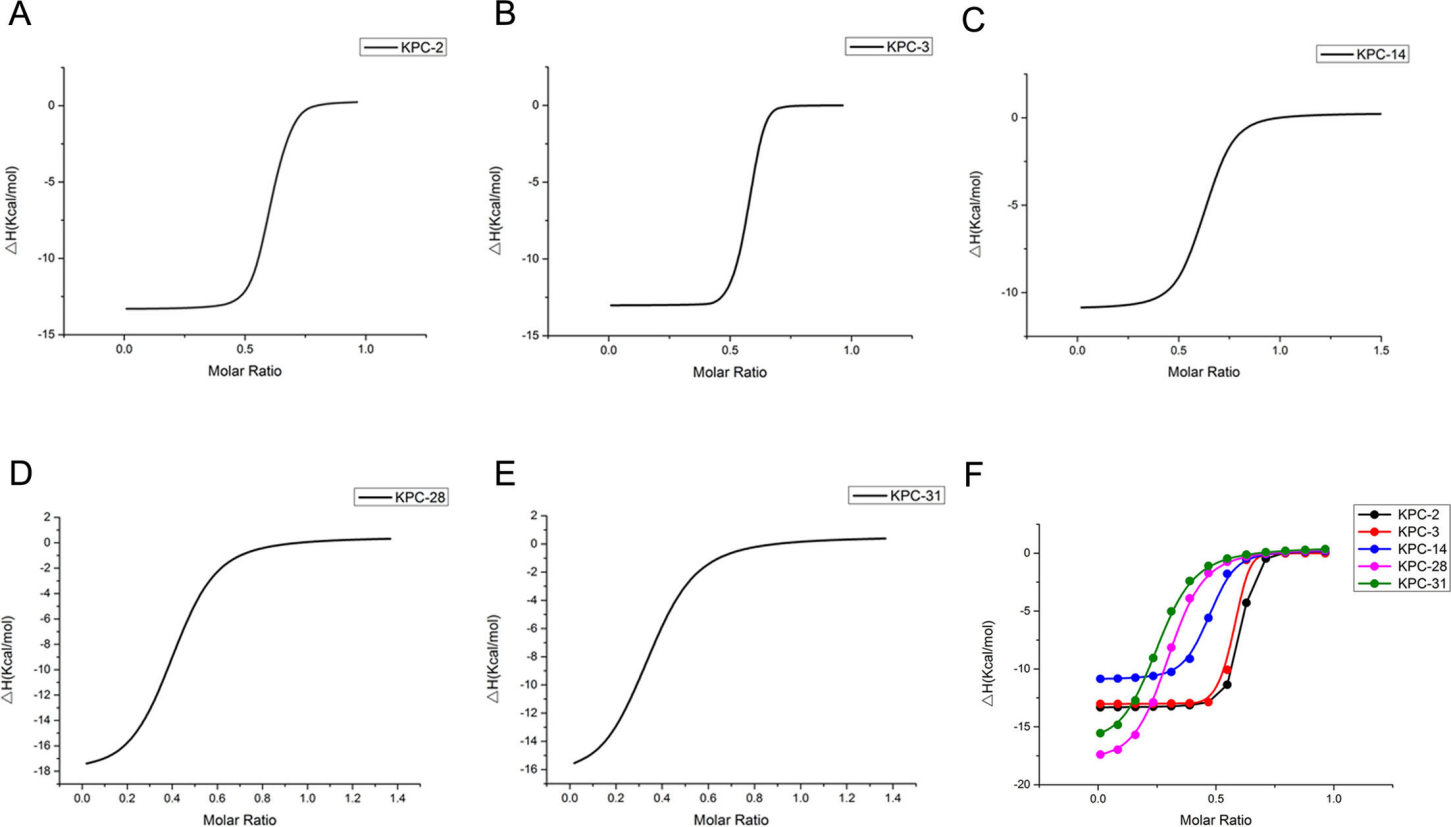
Examination of the interaction between AVI and KPC-2, KPC-3, KPC-14, KPC-28, and KPC-31 through ITC binding analysis. (F) A collection of images of data from panels A–E.

Models of KPC-3, KPC-14, KPC-28, and KPC-31 bound to AVI were generated by superimposing the KPC-2:AVI crystal structure (PDB: 5UJ3) onto energy-minimized models. These structural analyses revealed variations in ligand positioning within the active site and shifts in the β-hairpin loop conformation in the KPC-3 structures (Fig. 2). The 242–243 GT deletions facilitated hydrogen bond formation between the tyrosine hydroxyl and the AVI aminothiazole ring (Fig. 2), explaining the 3- and 12-fold increase in the dissociation constant (*K*_*D*_) value of AVI for KPC-14 and KPC-28, respectively, ultimately leading to increased MICs. Remarkably, AVI binding reduced ligand-protein interactions in both KPC-14 and KPC-28. In KPC-31–CAZ complexes, the β-hairpin loop conformation significantly altered to accommodate CAZ’s larger substituent. The D179Y substitution facilitated CAZ hydrolysis via an additional hydrogen bond between guanidinium nitrogen of arginine and the carboxyl group of the oxyimino moiety in CAZ (Fig. S4). Isothermal titration calorimetry (ITC) showed weaker KPC-14, KPC-28, and KPC-31 binding to AVI than KPC-2 or KPC-3 (Fig. 3). The *K*_*D*_ for KPC-31 (3.17 × 10^−6^ ± .752 × 10^−6^ M) with AVI was ~60-fold higher than KPC-2 (49.4 × 10^−9^ ± 22.9 × 10^−9^ M), explaining KPC-31’s CAZ-AVI resistance despite its low CAZ affinity (Table 5).

**TABLE 5:** The parameters of KPC-2, KPC-3, KPC-14, KPC-28, and KPC-31 to bind AVI presented by ITC and molecular docking

Complex of variants	Calculated value (*K*_*D*_)	Δ*H* (kcal/mol)	Binding energy of molecular docking (kcal/mol)
KPC-2-AVI	49.4 × 10^−9^ ± 22.9 × 10^−9^ M	13.6 ± 0.434	−6.0095
KPC-3-AVI	7.49 × 10^−9^ ± 3.05 × 10^−9^ M	13.0 ± 0.133	−6.0014
KPC-14-AVI	154 × 10^−9^ ± 34.3 × 10^−9^ M	11.2 ± 0.23	−5.9369
KPC-28-AVI	636 × 10^−9^ ± 220 × 10^−9^ M	18.9 ± 1.21	−5.6894
KPC-31-AVI	3,170 × 10^−9^ ± 752 × 10^−9^ M	17.8 ± 1.01	−5.9466

## DISCUSSION

An increasing number of KPC variants exhibit increased resistance to CAZ-AVI at the expense of carbapenem hydrolysis (11, 13–15). Recognizing the multifaceted nature of CAZ-AVI resistance, a comprehensive evaluation of clinical KPC variants over time becomes imperative for redefining therapeutic strategies against severe infections caused by KPC-producing strains. Phylogenetic ancestor reconstruction of KPC-2 to KPC-36 revealed two main clusters: KPC-2 and KPC-3 cluster (Fig. 1). The KPC-3 cluster showing higher CAZ MIC_50_ and MIC_90_ values for CAZ than KPC-2 cluster variants underscores increased resistance to CAZ. Six high-frequency mutation sites (P104, W105, D179, F207, V240, and T243; Fig. 1C) were identified in KPC variants 2–36; analysis of variants 37–245 revealed three additional sites: EL166-167, L169, and loops 261–277. EL166-167, L169, and D179 are located within the Ω-loop (R164-D179) (Fig. S5), crucial for the active site’s conserved salt bridge (16, 17). V240 and T243, in loops 237–243, are near the 234–236 KTG motif adjacent to the S70 active site (Fig. S5). The mutation region spanning sites 269–271, along with its adjacent region at sites 261–277, was dominated by insertion mutations, such as KPC-29, KPC-41, KPC-50, KPC-52, KPC-67, KPC-73, KPC-78, KPC-82, and KPC-97 (9, 15, 18, 19). We specifically characterized KPC-29, with 269–271 KDD insertions, displaying a lower MIC value for CAZ-AVI compared to KPC-3. However, clinical *K. pneumoniae* producing KPC-29 was reported to be resistant to CAZ-AVI (12). KPC variants harboring mutations at high-frequency sites, including P104, L169P, D179Y, V240G, 242–243 GT deletions, and 261–277 insertions, exhibit increased resistance to CAZ. These amino acid substitutions on the Ω-loop of class A β-lactamases are typically associated with expanded substrate specificity toward CAZ.

There were also some conflicting data about D179Y in these reports. For example, Tsivkovski and Lomovskaya suggested that KPC-2^D179Y^ (KPC-33) enhanced the hydrolysis capacity of CAZ (20), which is consistent with our results, while Compain and Arthur did not (6). The *K_i_* values of KPC-113 for AVI were all higher than those of KPC-2 (21), manifesting the attenuated inhibitory effects of the inhibitor on KPC-113, which is consistent with the trends we have observed. Tsivkovski and Lomovskaya reported that D179Y substitution causes a 20-fold increase in the 50% inhibitory concentration for inhibition of CAZ hydrolysis by AVI. In addition, the D179Y and L169P variants hydrolyze CAZ with 10- and 4-fold higher efficiencies, respectively, than that of wild-type KPC-2. Thus, microbiological and biochemical experiments implicate both a decreased ability of AVI to interact with KPC-2 variants and an increase in the efficiency of CAZ hydrolysis in resistance to CAZ-AVI (20). Mutations outside the classical Ω-loop (R164-D179), loops 237–243, and loops261–277 also have a role in enhancing CAZ-AVI resistance, such as KPC-71 with 182S insertion reported by Yu et al. (22), which is also confirmed by KPC-3^A125V^, KPC-3^A201V, V240G^, and KPC-3 ^P104R, A120L, G147K, A201V, V240G^ in our study, suggesting that mutants outside the three loops should not be ignored. These studies have demonstrated that specific mutations in KPC (CAZ-AVIR) can be induced by CAZ-AVI. In fact, the possibility that antibiotics may act not only as selectors for antibiotic-resistant bacteria but also as resistance promoters was also proposed by Blázquez et al. (23–25).

To gain insight into the selective forces driving KPC diversification, assess the impact of amino acid mutations within KPC on resistance to CAZ and CAZ-AVI, and understand how these mutations were favored under antibiotic exposure conditions, we engineered a set of 33 natural variants and 8 synthetic variants (Tables 1–3). Among the antibiotics evaluated, CAZ-AVI and piperacillin-tazobactam MICs displayed the most pronounced alterations in response to introduced amino acid changes. Specifically, CAZ-AVI MICs increased in 5 of 18 KPC-2 variants and 11 of 21 KPC-3 variants, including 8 non-natural variants. Piperacillin-tazobactam MICs decreased in 35 of 39 variants (Fig. S2). Some mutations enlarged the active site, favoring oxyimino cephalosporin interaction while impairing penicillin interaction, resulting in extended-spectrum β-lactamases (ESBL) phenotypes (19, 26). González et al. reported that in-cell kinetics and protein stability of β-lactamase may represent another molecular trait optimized in evolution, which is related to membrane fusion, protein secretion, and even protection of proteins from degradation (27).

In our study, the maximum decrease in protein levels was observed in the KPC-15 and KPC-28 variants, but all of them exhibited higher MICs for CAZ-AVI compared to KPC-3. KPC-35, carrying an L169P mutation, demonstrated improved catalytic efficiency for CAZ but at the cost of reduced catalytic efficiency against carbapenems compared to KPC-2. It is foreseeable that single amino acid substitution at the same site resulted in distinct resistance phenotypes, as seen with the L169M mutation (KPC-12), which conferred susceptibility to CAZ. Sometimes, both insertion and deletion at the same sites exhibited similar resistance; for example, KPC-3^166-167EL ins^ and KPC-3^166-167EL del^ decreased MICs against all tested antibiotics except for CAZ-AVI. Mutations in signal peptide regions (R6H/P, V8I, A18S; positions 1–29) had minimal effects on β-lactam resistance due to their distance from the active site, as confirmed by the similar resistance profiles of KPC-3^L10P, A177E, D179Y^ and KPC-3^A177E, D179Y^ (differing by L10P). In fact, signal peptides exported β-lactamase into the periplasm, and signal peptide mutation affected the fitness cost of *bla*_KPC_ plasmids (28, 29). No CAZ-AVI-resistant KPC variants exhibited signal peptide mutations, consistent with previous findings showing that signal peptide mutations generally reduce enzyme activity or have little effect (10, 12).

Of the eight non-natural variants that have emerged, lower MICs were detected in piperacillin, piperacillin-tazobactam, cefazolin, cefuroxime, imipenem, and meropenem. These variants, except for KPC-53 and KPC-66, cannot overcome the inhibition of these commonly used clinical drugs, so the likelihood of detection is greatly reduced. The imipenem (IPM) MICs of KPC-carrying *Escherichia* coli were higher than meropenem (MEM) in both clinically isolated (30, 31) and cloned strains (32–35), which is generally consistent with our report, and the same trend was observed in *Escherichia* coli without KPC, which seems to be related to the greater sensitivity of gram-negative bacteria to MEM (36). At the same time, some KPC variants have been observed to be more hydrolyzed to MEM (35; 37), which seems to be contrary to the results of antibiotic sensitivity tests. The bacterial environment *in vivo* is relatively complex; the permeability of the outer membrane and the expression of lactamase will affect the antibacterial activity of antibiotics.

Our findings suggest that CAZ plays a pivotal role in driving KPC diversification, selecting for variants that transition from poor to efficient CAZ hydrolysis. This effect is more pronounced for CAZ than other β-lactams in serine (38, 39) and metallo-β-lactamases (40) for two primary reasons: first, β-lactams poorly hydrolyzed by a given β-lactamase are more likely to drive diversification; second, the evolving epidemiological context, particularly antibiotic usage patterns, plays a crucial role. Antibiotic consumption is a major driver of antibiotic resistance, and in this regard, exposure to CAZ, whether concurrently or sequentially, in clinical settings or environmental contexts may have facilitated secondary diversification and dissemination of KPC variants. We attempted to elucidate the reduced hydrolysis of CAZ by KPC-31 and the simultaneous increase in the MIC of CAZ-AVI. This phenomenon is in part attributed to the diminished inhibitory effect of AVI on KPC-31, a revelation not previously highlighted in the literature to the best of our knowledge. KPC variants enhance affinity to CAZ while weakening affinity to AVI by inducing structural changes. However, present studies still focus on the interaction between CAZ and KPC, lacking evidence confirming the weakened affinity between AVI and KPC. Here, we utilized ITC to explore the interaction between KPC and AVI. The ability of KPC-31 (3.17 × 10^−6^ ± 0.752 × 10^−6^ M) to bind AVI is significantly weaker than that of KPC-2 (49.4 × 10^−9^ ± 22.9 × 10^−9^ M) (Fig. 3).

### Conclusions

In conclusion, our comprehensive study sheds light on the intricate dynamics of KPC diversification and β-lactam resistance. The emergence and dissemination of KPC variants present a formidable challenge in the clinical management of infections caused by these multidrug-resistant pathogens. KPC-2 cluster variants exhibiting elevated CAZ-AVI MICs (KPC-4, KPC-5, KPC-6, and KPC-14), except for KPC-25, are accompanied by varying degrees (4- to 32-fold) of increased CAZ MICs. In contrast, the majority of KPC-3 cluster variants with elevated CAZ-AVI MICs (11 out of 12) maintain a relatively stable CAZ MIC (between 0.5- and 2.0-fold), with only KPC-3^A201V, V240G^ showing a fourfold increase in CAZ MIC. This suggests that the underlying mechanisms causing elevated CAZ-AVI MICs in KPC-2 and KPC-3 cluster variants are different. For the former, increased CAZ hydrolysis is strongly implicated, while for the latter, diminished AVI inhibition is likely the primary cause. Our findings underscore the significant role of CAZ and AVI as two driving forces behind the evolution of KPC, serve as a foundational step in this endeavor, and contribute to our understanding of the mechanisms underpinning antibiotic resistance in KPC-producing bacteria. Antibiotics may act not only as selectors for antibiotic-resistant bacteria but also as resistance promoters.

## MATERIALS AND METHODS

### Phylogenetic analysis

The nucleotide sequences of 34 *bla*_KPC_ genes, ranging from *bla*_KPC-2_ to *bla*_KPC-36_, were retrieved from the Beta-Lactamase Database—Structure and Function website (http://www.bldb.eu/Enzymes.php) and presented in Table S1. These nucleotide sequences were translated and aligned using the ClustalW algorithm implemented in MEGA, followed by manual correction of any sequencing and alignment errors. Subsequently, both ML and network phylogenetic trees were constructed, with branch support calculated based on 500 bootstrap replicates (41).

### Antimicrobial susceptibility testing

The *bla*_KPC_ amplicons (full length) were cloned into the Tn4401a-pET28a plasmid between the *BamH*I (as the 5′ end upstream) and *Xba*I (as the 3′ end downstream) restriction sites, which is the opposite direction of conventional induced expression for removing the His-tag expression. This insertion was downstream of the *bla*_KPC_ *Tn*4401a promoter. The *Tn*4401a promoter was inserted between the *EcoR*I and *BamH*I sites. KPC expression was achieved by inserting the natural promoter Tn4401a upstream of the *bla*_KPC_ gene, thus avoiding the use of the pET-28a vector’s T7 promoter and the resulting His-tag fusion. *E. coli* DH10B strains containing the generated KPCs-Tn4401a-pET28a plasmid underwent antimicrobial susceptibility testing in triplicate using the standard broth microdilution, following the Clinical and Laboratory Standards Institute (CLSI) recommendations (CLSI M100-S34) . *Klebsiella pneumoniae* expressing KPC-3 and *E. coli* DH10B containing an empty plasmid served as positive and negative controls, respectively. AVI was tested in a twofold dilution with ceftazidime or aztreonam, maintaining a fixed concentration of 4 mg/L. Tazobactam was assessed in combination with twofold dilutions of piperacillin at a fixed concentration of 4 mg/L. The 2024 CLSI standards were applied to interpret the MIC values.

### Multistep mutant selection of CAZ-AVI

The *E. coli* DH10B strains containing the generated KPC-3-Tn4401a-pET28a plasmid were cultured overnight in 5 mL Luria-Bertani (LB) broth. Approximately 10^9^ CFU was added to the 5 mL LB broth containing CAZ-AVI. We carried out 10 groups of tests simultaneously with different concentrations of AVI at 1–512 mg/L. The endpoint of the test is the CAZ reaching 512 mg/L or the induction days reaching 30 days. At the same time, parental strain was passaged without drugs as a control. We saved all the strains that appeared during the induction process and performed DNA sequencing.

### Protein purification

For protein production, the *bla*_KPC_ gene (excluding the signal peptide, 1–29 AA) was inserted into the pET28a plasmid. The primers used for site-directed mutagenesis and peptide signal elimination are provided in Table S2. The recombinant KPC_30–293_-pET28a plasmids were introduced into *E. coli* BL21 Rosetta-gami DE3 for inducing protein expression with 0.2 mM isopropyl-β-d-thiogalactoside. The soluble protein fraction of cellular supernatant lysed via sonication was then applied to a HisTrapTM HP column for protein purification. The His-tag was removed by thrombin protease cleavage. Finally, for desalination and imidazole removal, the eluted protein was loaded into a dialysis bag and dialyzed overnight. SDS-PAGE analysis confirmed that the purity of proteins and the protein concentrations were determined using the Pierce BCA Protein Assay Kit.

### Kinetic parameters in steady state

The kinetic parameters of KPCs hydrolyzing β-lactams were determined using purified KPC enzymes in 100 mM sodium phosphate buffers (pH 7.0) at 37℃ by a SHIMADZU UV2550 spectrophotometer. The initial velocities vs substrate concentrations were measured at least thrice. The molar extinction coefficients for tested substrates were obtained from a previous study (33, 42). The *k*_cat_ and *K*_*m*_ values were calculated with GraphPad Prism version 8.4 using non-linear least square fit of the data to the Michaelis-Menten equation: *v* = *k*_cat_ [S] / (*K*_*m*_ + [S]) (5, 33, 43). Consequently, for ceftazidime rather stable to the action of the KPC, progress curves were fitted to the equation *S_0_*/*v* = (*K*_*m*_ + *S_0_*) / *V*, where *S_0_* << *K*_*m*_ to determine the *k*_cat_/*K*_*m*_ ratios, which reflect the catalytic efficiency of KPC enzymes against the substrate.

### Molecular modeling

The KPC-2 crystal structure (PDB code 5UJ3) served as the template for molecular modeling. The homology model was built for KPC-3/KPC-14/KPC-28/KPC-31 using MODELLER. Three-dimensional structures of the ligands were generated using CORINA. To predict the conformation of amino acid side chains, the Dunbrack backbone-dependent rotamer library was utilized, based on the protein’s global energy minimum. Prepared protein systems were further checked by Ramachandran plots. To predict the Michaelis-Menten complex, the Autodock Vina docking method was employed, as previously described (42). Covalent docking calculations were carried out using the GOLD Suite version 5.2 and the GoldScore scoring function. The acyl-enzyme complex was formed by covalently connecting the OG atom of the Ser70 residue to the open form of β-lactam ring. The model with CAZ or AVI in the binding conformation, where β-lactam carbonyl was directed into the oxyanion hole and exhibited the most favorable hydrogen bond and hydrophobic interactions, was selected for further analysis. Molecular modeling images were generated using UCSF CHIMERA.

### ITC assays

Purified KPC_30–293_ proteins and drugs were prepared in a buffer containing 300 mM NaCl and 5 mM sodium phosphate at pH 7.0. High-sensitivity ITC experiments were conducted in a controlled environment using the MicroCal PEAQ-ITC200 system at a temperature of 25°C. The affinity parameters were calculated using the MicroCal PEAQ-ITC200 analysis software (44).

## SUPPLEMENTAL MATERIAL

Supplemental material msystems.00184-25-s0001.pdf Supplemental figures and tables.

ASM does not own the copyrights to Supplemental Material that may be linked to, or accessed through, an article. The authors have granted ASM a non-exclusive, world-wide license to publish the Supplemental Material files. Please contact the corresponding author directly for reuse.
