# Supplementary material for: Diverse evolutionary trajectories of Klebsiella pneumoniae carbapenemase: unraveling the impact of amino acid substitutions on β-lactam susceptibility and the role of avibactam in driving resistance
Source: mSystems. 2025 Mar 11;10(4):e00184-25. doi: 10.1128/msystems.00184-25 (PMC12013264; doi:10.1128/msystems.00184-25)
Supplement: Supplemental material — Supplemental figures and tables. [file msystems.00184-25-s0001.pdf]

Wei et al., Fig. S1.

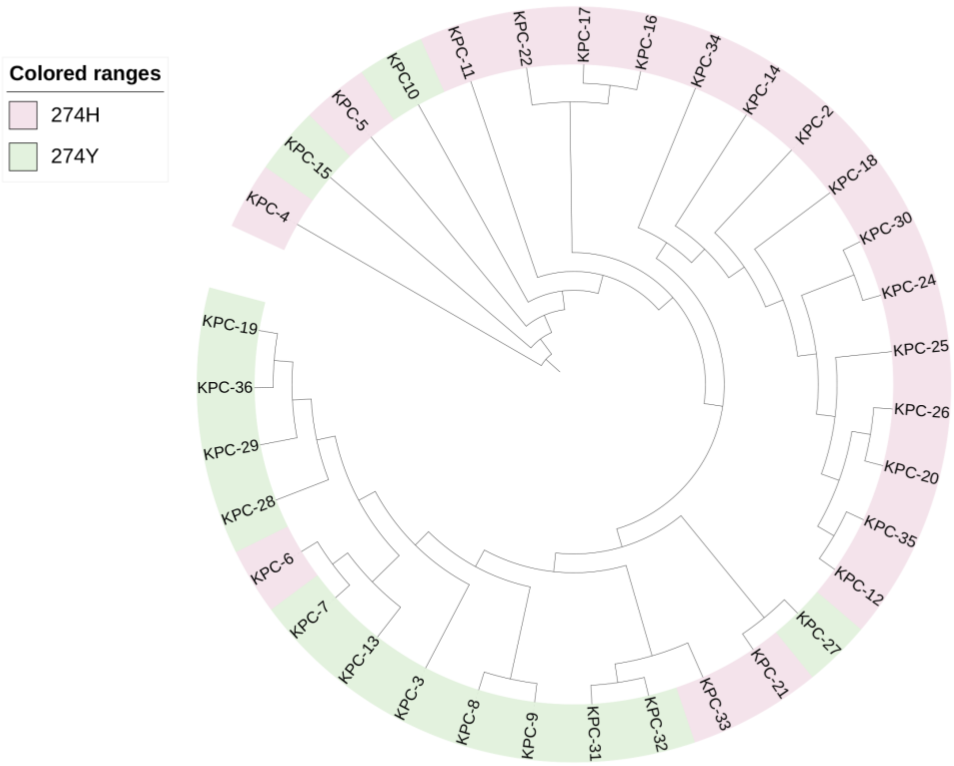

Figure S1 Maximum-likelihood (ML) phylogenetic tree of 44 KPC variants

The sequences from *bla*$_{KPC-2}$ to *bla*$_{KPC-36}$ were obtained from the Beta-Lactamase Data Base-Structure and Function website (http://www.bldb.eu/BLDB.php?prot=A#KPC). The construction of the maximum-likelihood phylogenetic tree was carried out using the MEGA-X (Molecular Evolutionary Genetics Analysis) program and subsequently modified using the iTOL tool. Support for the relevant nodes was estimated through bootstrapping with 1000 replicates, resulting in the differentiation of two distinct phylogroups corresponding to KPC-2 (274H) and KPC-3 (274Y).

Wei et al., Fig. S2.

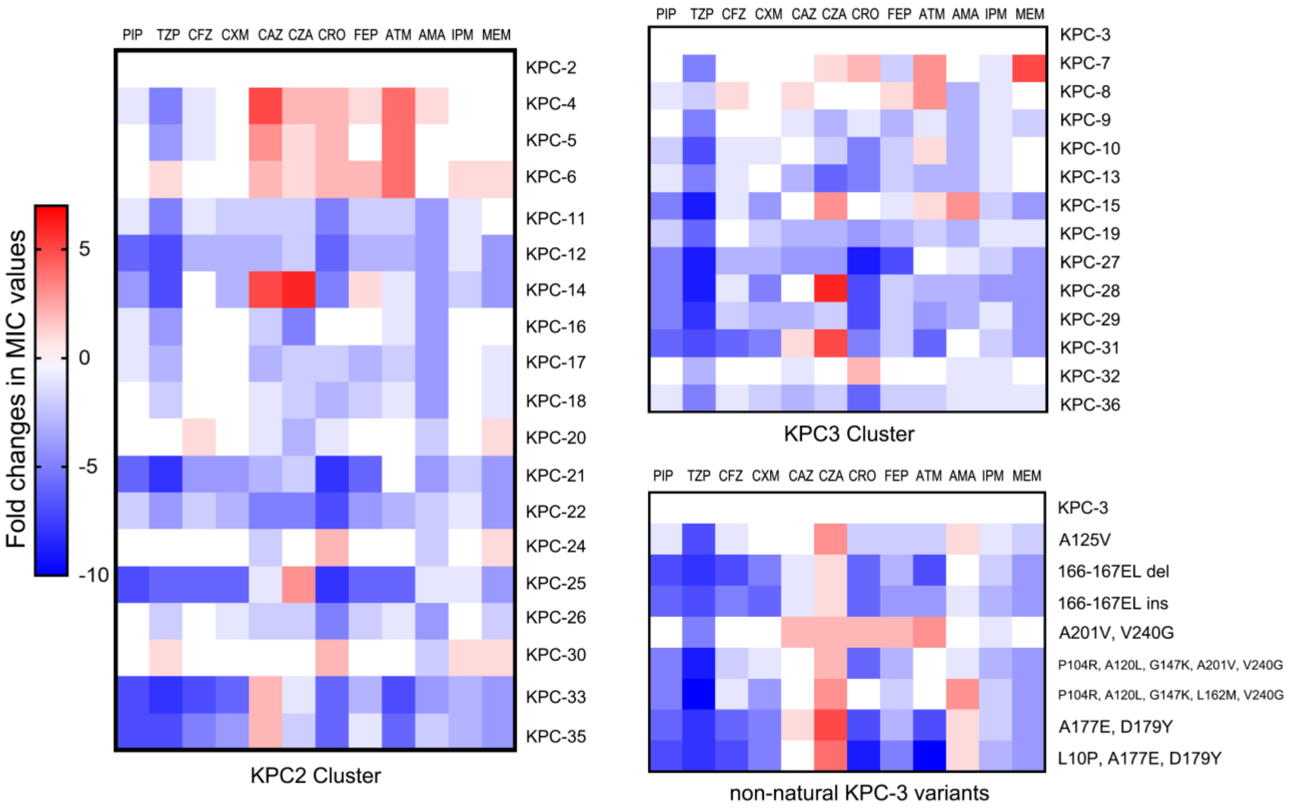

Figure S2 Heat maps depicting the log2 fold changes in MIC values of a panel of antibiotics representing three structural classes of β-lactams

The log2 fold change in MIC compared to the parent family member is represented by the heat map intensity scale. *The MICs are the medians of three or more values obtained or an MIC value obtained multiple times. PIP, piperacillin; TZP, piperacillin-tazobactam; CZO, cefazolin; CXM, cefuroxime; CAZ, ceftazidime; CZA, ceftazidime-avibactam; CRO, ceftriaxone; FEP, cefepime; ATM, aztreonam; AMA; aztreonam-avibactam; IPM, imipenem; MEM, meropenem.

Wei et al., Fig. S3.

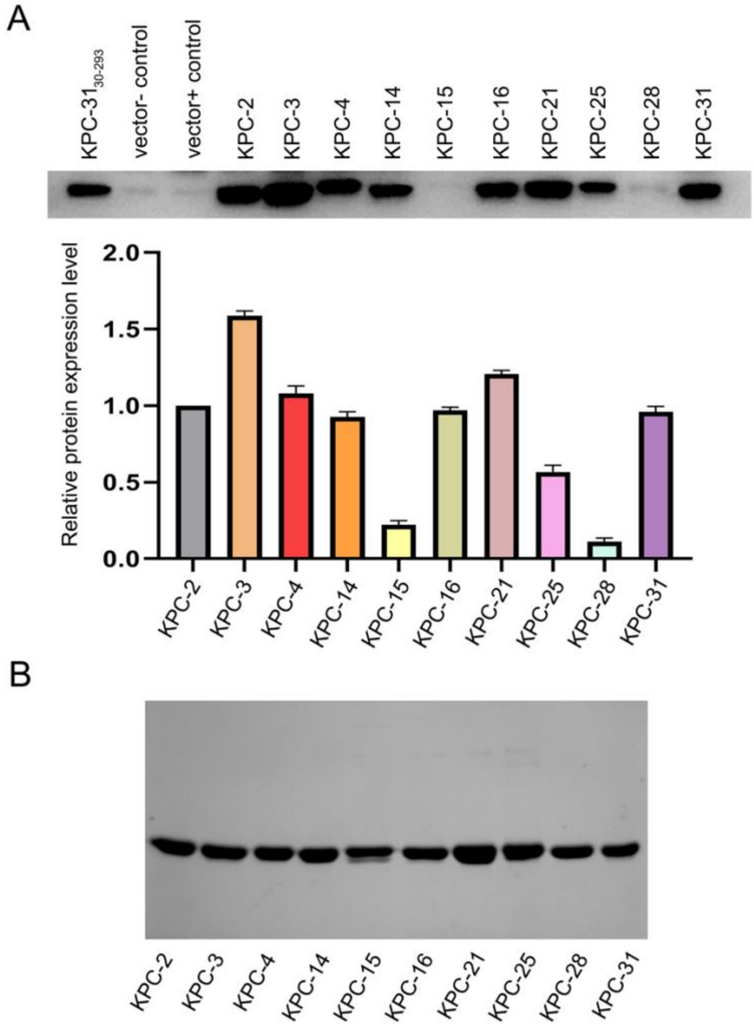

Figure S3 Expression of various KPC variants

A. Immunoblot depicting the investigated KPC variants (KPC-2, -3, -4, -14, -15, -16, -21, -25, -28, -31); membranes were prepared and exposed concurrently, following identical conditions. Purified KPC-31$_{30-293}$ protein served as a constitutively expressed control. Notably, the KPC-3 variant exhibited the highest expression level, whereas KPC-15, -25, and -28 variants displayed significantly lower expression levels compared to the others. **B**. Evaluation of the purity of the ten purified KPC variants via SDS-PAGE analysis.

Wei et al., Fig. S4.

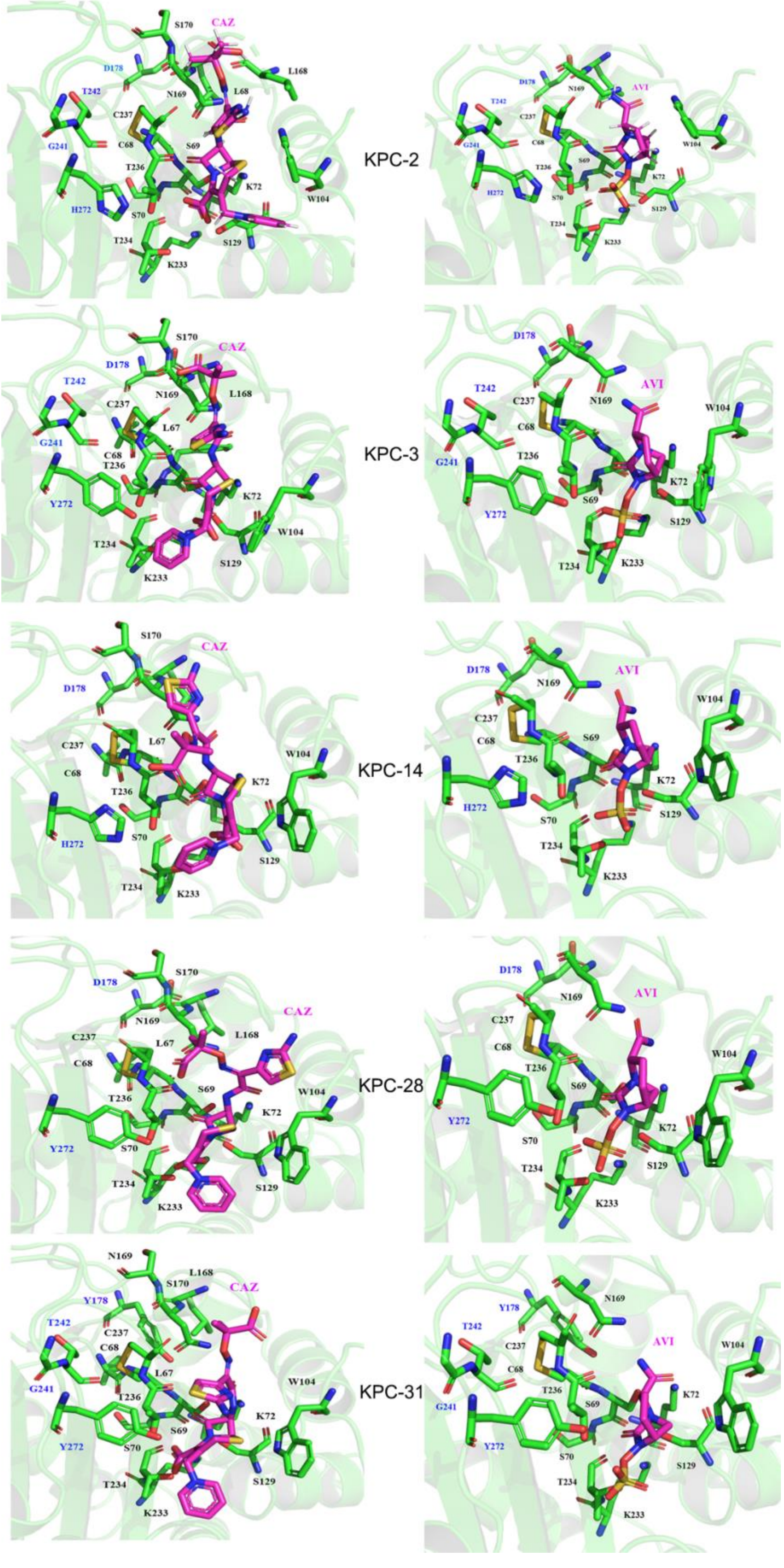

Supplementary Figure S4: Models of interaction between KPC-2, -3, -14, -28 and -31 with CAZ (left panel) or AVI (right panel).

Wei et al., Fig. S5.

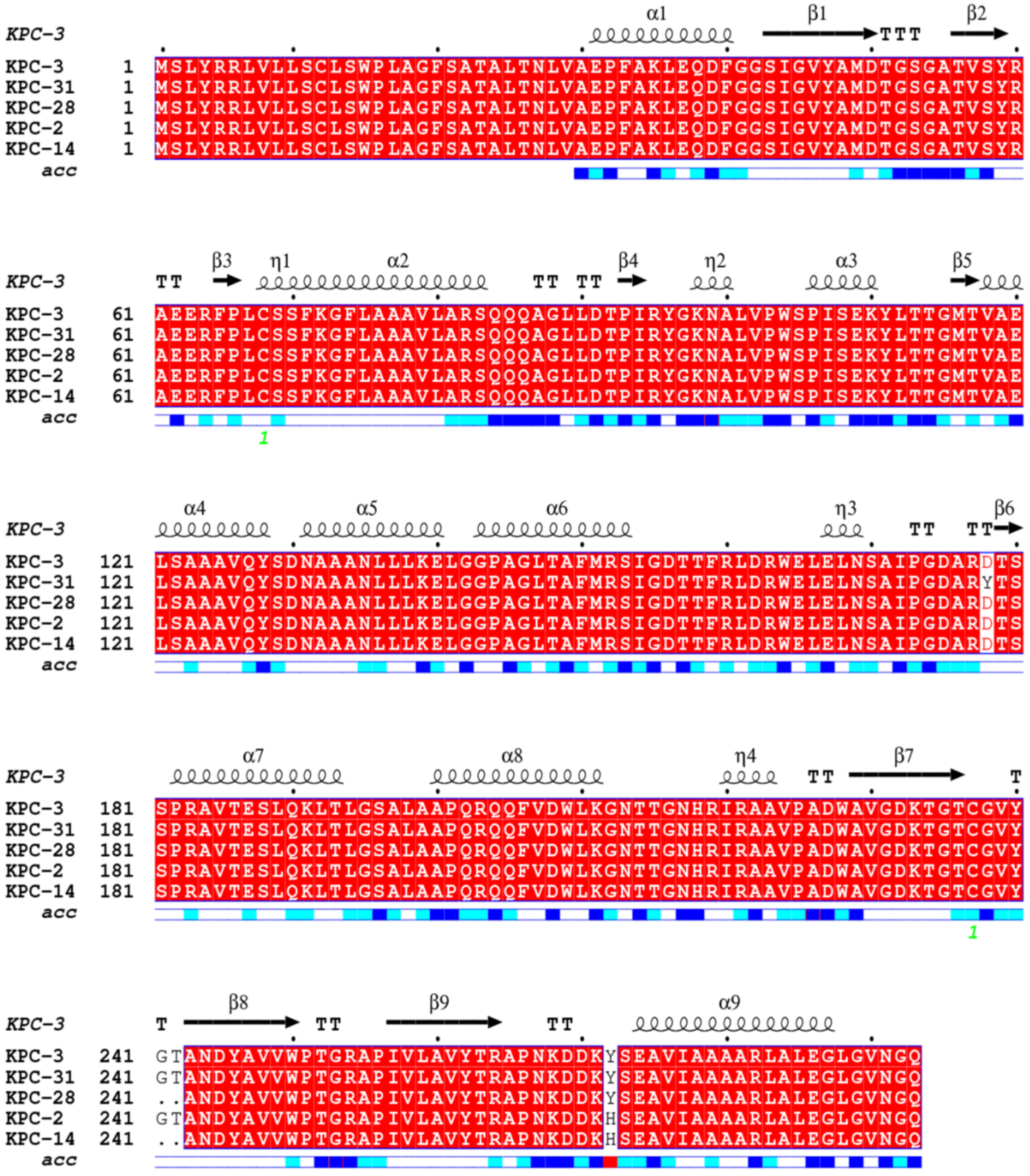

Supplementary Figure S5: Alignment of the amino acid sequence of KPC-3 with that of KPC-2, KPC-14,

KPC-28, and KPC-31. Alpha helices are indicated by helix loop lines and β strands by continuous arrow lines. Ellipses indicate the gaps inserted to optimize the alignment.

Table S1 GeneBank ID of KPC variants

**Variant**	**GenBank ID**	**Country**	**Year**
KPC-1/2	**LDDY01000008**	USA	2001
KPC-3	**KJ748372**	USA	2004
KPC-4	**JMUK01000029**	Scotland	2004
KPC-5	**LPOR01000022**	Puerto Rico	2008
KPC-6	**EU555534**	Puerto Rico	2008
KPC-7	**EU729727**	USA	2008
KPC-8	**FJ234412**	Puerto Rico	2008
KPC-9/23	**MH450213**	Israel	2009
KPC-10	**GQ140348**	Puerto Rico	2010
KPC-11	**HM066995**	USA	2010
KPC-12	**HQ641422**	china,ZJ	2010
KPC-13	**HQ342889**	Thailand	2012
KPC-14	**JX524191**	New York City	2012
KPC-15	**KC433553**	Taiwan	2013
KPC-16	**KC465199**	Taiwan	2013
KPC-17	**KC465200**	Taiwan	2015
KPC-18	**KT884517**	USA	2014
KPC-19	**KJ775801**	unknown	2009
KPC-20	**MF772496**	Israel	2014
KPC-21	**LN609376**	Portugal	2014

KPC-22	**KM379100**	Southern Taiwan	2009
KPC-24	**KR052099**	Chile: Temuco	2015
KPC-25	**KU216748**	United States	2015
KPC-26	**KX619622**	Brazil: Rio de Janeiro	2016
KPC-27	**KX828722**	unknown	2016
KPC-28	**KY282958**	unknown	2016
KPC-29	**KY563764**	USA	2017
KPC-30	**KY646302**	Brazil	2017
KPC-31	**MAPH01000113**	USA	2016
KPC-32	**MAPO01000050**	USA	2016
KPC-33	**CP025144**	USA: New York	2017
KPC-35	**MH404098**	USA: California	2018
KPC-36	**MH593787**	Italy	2018

Table S2 primer of directed PCR

BamH1-KPC-F	ATCGGATCCGCGGAACCATTCGCTAAACT
EcoR1-KPC-R	ATCGAATTCTTACTGCCCGTTGACGCCCA
KPC-2 to KPC-20-F1:	GCTGACCAACCTCATCGCGGAACCATTCGC
KPC-2 to KPC-20-R1:	GCGAATGGTTCCGCGATGAGGTTGGTCAGC
KPC-5 to KPC-11-F1:	GCAAAAATGCGCTGGTTCTGTGGTCACCCATC
KPC-5 to KPC-11-R1:	GATGGGTGACCACAGAACCAGCGCATTTTTGC
KPC-3 to KPC-9-F1:	CCGGAACCTGCGGAGCGTATGGCACGGC
KPC-3 to KPC-9-R1:	GCCGTGCCATACGCTCCGCAGGTTCCGG

KPC-21 to KPC-19-R:	CTAGTCTAGATTACTGCCCCGTGACGCCCAATCCCTCGAG
KPC-3 to KPC-29-F1:	CGCCTAACAAGGATGACAAGGATGACAAGTACAGCGAGGCCGTCATCGC
KPC-3 to KPC-29-R1:	GCGATGACGGCCTCGCTGTACTTGTCATCCTTGTCATCCTTGTTAGGCG
KPC-3 to KPC-12-F1:	CAGGCCGGCTTGCTGGGCACACCCATCCGTTAC
KPC-3 to KPC-12-R1:	GTAACGGATGGGTGTGCCCAGCAAGCCGGCCTG
KPC-12 to KPC-13-F1:	GTTCCGTCTGGACTGCTGGGAGCTGGAGCTG
KPC-12 to KPC-13-R1:	CAGCTCCAGCTCCCAGCAGTCCAGACGGAAC
KPC-12 to KPC-13-R:	CTAGTCTAGATTACTGCCCGTTGACGCCCAATCCCTCGAGCGCGAGTCCAGC CGCAGCG
KPC-31 to KPC-32-F1:	CGGAGTGTATGGCATGGCAAATGACTATGCCG
KPC-3 1to KPC-32-R1:	CGGCATAGTCATTTGCCATGCCATACACTCCG
KPC-31 to KPC-33-R:	CTAGTCTAGATTACTGCCCGTTGACGCCCAATCCCTCGAGCGCGAGTCTAGC CGCAGCGGCGATGACGGCCTCGCTGTGCTTGTC
KPC-2 to KPC-12 F	TGGGAGCTGGAGATGAACTCCGCC
KPC-2 to KPC-12 R	GGCGGAGTTCATCTCCAGCTCCCA
KPC-15 F	ACGGCCTTCATGCGCTCTATC
KPC-15 R	GATAGAGCGCATGAAGGCCGT
KPC-13 -507c to KPC-13 F	TGAACTCCGCTATCCCAGGCGATG
KPC-13 -507c to KPC-13 R	CATCGCCTGGGATAGCGGAGTTCA
KPC-17 to KPC-16 F2	TGGATACCGGTTCAGGCGCAAC
KPC-17 to KPC-16 R2	GTTGCGCCTGAACCGGTATCCA
KPC-17 to KPC-22 F	GCGCTGGTTCCGGGGTCACCCATCTCGG
KPC-17 to KPC-22 R	CCGAGATGGGTGACCCCGGAACCAGCGC
KPC-2 to KPC-35 F	ACCGCTGGGAGCTGGAGCCGAACTCCGCCATCCCA
KPC-2 to KPC-35 R	TGGGATGGCGGAGTTCGGCTCCAGCTCCCAGCGGT
KPC-3 to KPC-36 F	ATACCACGTTCCGTCTGGAACGCTGGGAGCTGGAG

KPC-3 to KPC-36 R	CTCCAGCTCCCAGCGTTCCAGACGGAACGTGGTAT

Table S3 MIC_{50} and MIC_{90} of various antibiotics in the context of KPC-2 and KPC-3 cluster variants

		PIP	TZP	CZO	CXM	CAZ	CAZ-AVI	CRO	FEP	ATM	ATM-AVI	IPM	MEM
	KPC-2	>256	256	256	>256	8	0.25	128	4	32	0.125	1	0.25
	KPC-3	>256	>256	256	>256	64	0.5	128	8	64	0.0625	2	0.25
MIC_{50} (mg/ L)	KPC-2 cluster	256	16	256	256	2	0.0625	4	1	16	<0.0156	1	0.0625
	KPC-3 cluster	128	4	128	128	64	0.5	2	2	8	0.0312	1	0.0625
	KPC cluster	256	8	128	256	32	0.25	4	2	16	0.0312	1	0.0625
MIC_{90} (mg/ L)	KPC-2 cluster	>256	256	256	>256	8	0.25	128	4	32	0.0312	1	0.5
	KPC-3 cluster	>256	16	256	>256	128	4	>256	4	128	0.125	1	0.25
	KPC cluster	>256	256	256	>256	128	4	>256	8	>256	0.125	1	0.5

Count (%)	R	24(58.6)	7(17.1)	32(78)	33(80.5)	22(53.7)	4(9.8)	26(63.4)	3(7.4)	26(63.4)	0(0)	0(0)	1(2.4)
	I	1(2.4)	4(9.7)	0(0)	5(12.2)	6(14.6)	0(0)	7(17.1)	11(26.8)	4(9.8)	0(0)	3(7.3)	0(0)
	S	16(39)	30(73.2)	9(22)	3(7.3)	13(31.7)	37(90.2)	8(19.5)	27(65.8)	11(26.8)	41(100)	38(92.7)	40(97.6)

*The MICs are the medians of three or more values obtained or an MIC value obtained more than once. PIP, piperacillin; TZP, piperacillin-tazobactam; CZO, cefazolin; CXM, cefuroxime; CAZ, ceftazidime; CAZ-AVI, ceftazidime-avibactam; CRO, ceftriaxone; FEP, cefepime; ATM, aztreonam; ATM-AVI; aztreonam-avibactam; IPM, imipenem; MEM, meropenem.
